# Utilization of Percutaneous Mechanical Circulatory Support Devices in Cardiogenic Shock Complicating Acute Myocardial Infarction and High-Risk Percutaneous Coronary Interventions

**DOI:** 10.3390/jcm8081209

**Published:** 2019-08-13

**Authors:** Rabea Asleh, Jon R. Resar

**Affiliations:** Division of Cardiology, Department of Medicine, Johns Hopkins University School of Medicine, Baltimore, MD 21205, USA

**Keywords:** mechanical circulatory support, percutaneous coronary intervention, cardiogenic shock, acute myocardial infarction, outcome, patient selection

## Abstract

Given the tremendous progress in interventional cardiology over the last decade, a growing number of older patients, who have more comorbidities and more complex coronary artery disease, are being considered for technically challenging and high-risk percutaneous coronary interventions (PCI). The success of performing such complex PCI is increasingly dependent on the availability and improvement of mechanical circulatory support (MCS) devices, which aim to provide hemodynamic support and left ventricular (LV) unloading to enable safe and successful coronary revascularization. MCS as an adjunct to high-risk PCI may, therefore, be an important component for improvement in clinical outcomes. MCS devices in this setting can be used for two main clinical conditions: patients who present with cardiogenic shock complicating acute myocardial infarction (AMI) and those undergoing technically complex and high-risk PCI without having overt cardiogenic shock. The current article reviews the advancement in the use of various devices in both AMI complicated by cardiogenic shock and complex high-risk PCI, highlights the available hemodynamic and clinical data associated with the use of MCS devices, and presents suggestive management strategies focusing on appropriate patient selection and optimal timing and support to potentially increase the clinical benefit from utilizing these devices during PCI in this high-risk group of patients.

## 1. Introduction

Recent advances in percutaneous coronary intervention (PCI) technologies, including mechanical circulatory support (MCS) devices, have facilitated treatment of high-risk patients with complex coronary artery disease (CAD) and low left ventricular (LV) systolic function as well as patients with acute myocardial infarction (AMI) complicated by cardiogenic shock. These high-risk patients would otherwise be poor candidates for coronary artery bypass grafting (CABG) due to high surgical morbidity and mortality risks, and the ability to provide adequate hemodynamic support using MCS devices would potentially enable safer PCI and improve outcomes as compared to unprotected PCI strategy, surgical revascularization, or medical therapy alone [1]. However, despite the preemptive improvement in hemodynamics with the use of MCS devices [2], randomized trials using intraaortic balloon pump (IABP) or Impella 2.5 devices have not demonstrated significant reduction in mortality during high-risk and complex PCI as compared to unprotected PCI [3,4]. A clinical benefit from the use of MCS in the setting of cardiogenic shock complicating AMI has also not yet been conclusively demonstrated [5,6]. Besides possible methodological flaws in these trials, other important factors might have contributed to the lack of benefit seen in these studies and should be taken into consideration, such as inadequate hemodynamic support, inappropriate patient selection, and deferred or inappropriate timing of device insertion during the course of cardiogenic shock and also in relation to PCI.

In light of the development of new generation MCS devices with greater hemodynamic support and lower device-associated complications combined with careful planning and optimal timing of device utilization, a growing body of data is suggestive of improvement in procedural success and clinical outcomes [7,8], and thus opens the door for future research in this field to examine the benefit of optimal MCS use in the setting of high risk PCI among a highly selective group of patients. Herein, we provide the most updated data available on MCS in two different situations involving high risk patients undergoing PCI: (1) high risk patients without cardiogenic shock undergoing complex PCI, and (2) patients with AMI complicated by cardiogenic shock.

## 2. High-Risk Percutaneous Coronary Interventions

Over the last few decades, there has been a tremendous progression in coronary interventional techniques that enables performance of PCI in complex coronary lesions (heavily calcified and type C) that would previously not have been amenable to intervention. This includes improved guide catheters and wires, mother-child guide catheters, low-profile balloons, coronary atherectomy devices, dedicated chronic total occlusion devices and algorithms, and superior stent designs that enhance deliverability to achieve complete multivessel revascularization including those involving chronic totally occluded coronary vessels. However, each aspect of PCI, beginning from guide catheter engagement and ending with balloon inflation and stent deployment, is associated with potential risk of vascular damage and impairment of myocardial perfusion. For instance, patients at advanced age, with increased comorbidities, and underlying left ventricular dysfunction, may pose a clinical challenge, as complex PCI among these patients may incur a substantial risk that overweighs any benefit achieved from revascularization [4]. On the other hand, utilization of PCI even in older adults with AMI and cardiogenic shock has been shown to be associated with substantial reduction in mortality in a recent contemporary analysis involving older adults ≥75 years of age [9]. Although clinical judgment is important, one should not, therefore, exclude patients from PCI solely based on advanced age in the absence of clear contraindications.

Among high-risk patients with active ischemia, the need and the type of revascularization should be discussed by Heart Team in an individual base. The recommendation with respect to the type of revascularization (PCI versus CABG) should be generally guided by important criteria including the predicted surgical mortality (based on the Society of Thoracic Surgeons (STS) score), the anatomical complexity of CAD (based on the SYNTAX (Synergy between Percutaneous Coronary Intervention with Taxus and Cardiac Surgery) score), and the anticipated completeness of revascularization. The risks of periprocedural complications should be weighed up against the anticipated improvement in quality of life and long-term freedom from death, MI, and repeat vascularization for electing whether conservative therapy, PCI, or CABG is the recommended strategy. According to the current ESC guidelines [10], when suitable coronary anatomy for both procedures and low predicted surgical mortality exist, patients with three-vessel disease and diabetes in particular achieve greater benefit from CABG than PCI regardless of the SYNTAX score, while in patients without diabetes CABG is favored over PCI only when SYNTAX score is intermediate or high (>22). In the presence of significant left main CAD, CABG is preferred for patients with SYNTAX score >22 irrespective of the diabetic state. However, in the presence of complex CAD anatomy (i.e., unprotected left main CAD or three-vessel disease) and high surgical mortality (i.e., previous cardiac surgery, severe comorbidities, and frailty) precluding CABG, high-risk PCI with MCS protection may be suggested as an alternative strategy for achieving complete revascularization safely in this high-risk group of patients. Although high-risk PCI has not been well defined, it can be generally categorized into three major groups based on patient characteristics, lesion characteristics, and clinical presentation [2,11] (Table 1). Patient characteristics include increased age, comorbidities (such as diabetes mellitus, chronic kidney disease, and chronic obstructive lung disease), reduced left ventricular systolic function, and prior myocardial infarction [12,13,14]. Lesion characteristics include anatomical and procedural variables that determine the complexity of PCI from the technical perspectives and the potential risk of complications. These include PCI of unprotected left main stenosis, bifurcation disease, heavily calcified lesions, saphenous vein grafts, and chronic total occlusions [15,16]. Finally, the clinical characteristics, among which acute coronary syndrome presentation and heart failure symptoms are important elements to take into consideration when assessing PCI risk [17]. An example of a high-risk PCI that can be facilitated by MCS is in an elderly patient with comorbidities who presents acutely with reduced LV systolic function and has a heavily calcified left main bifurcation or three-vessel coronary artery disease.

As high-risk PCI is associated with increased risk of myocardial ischemia and hemodynamic compromise that may lead to circulatory collapse, the purpose of MCS is to diminish myocardial oxygen consumption and provide adequate cardiac output and myocardial perfusion during the procedure. Use of appropriate MCS devices allows adequate time to safely perform complex PCI with optimal results in these high-risk patients who would not otherwise tolerate complete revascularization [18]. It is critical, therefore, that MCS device insertion in this setting is performed prior to PCI as this enables confident proceeding with revascularization without the risk of circulatory collapse that may subsequently require emergent bailout MCS implementation. Although several patient- and lesion-specific variables are well-recognized predictors of adverse outcomes after PCI, a risk score to assess the need for MCS during PCI has not yet been developed and warrants further research. Despite the lack of a risk calculator, most interventional cardiologists would now consider the use of MCS devices in patients with severely reduced LV systolic function and complex coronary artery disease involving a large territory (such as sole-remaining vessel, unprotected left main, or three-vessel disease) [11].

## 3. Cardiogenic Shock Complicating Acute Myocardial Infarction

Cardiogenic shock is defined as the combination of sustained systemic tissue hypoperfusion and decreased cardiac output despite adequate circulatory volume and LV filling pressure. Specific clinical and hemodynamic criteria that define cardiogenic shock include systolic blood pressure of <90 mmHg for >30 min, cardiac index <2.2 L/min/m^2^ with hemodynamic support or <1.8 L/min/m^2^ without support, pulmonary capillary wedge pressure (PCWP) >15 mmHg, and evidence of end-organ damage (such as urinary output <30 mL/h, high lactate levels, or cool extremities) [19,20]. Cardiogenic shock can develop because of various pathologies that affect the heart with AMI involved in the majority of cases. It occurs in 6–10% of patients with AMI and remains a leading cause of death with in-hospital mortality exceeding 50% despite the implementation of guideline-directed medical therapy and early myocardial reperfusion by primary PCI [21,22]. Consequently, patients with cardiogenic shock complicating MI represent a high-risk group of patients with compromised cardiac function and hemodynamics who are more susceptible to circulatory collapse during PCI [23]. Indeed, even though a substantial improvement in PCI techniques and pharmacology has occurred over time, this has not translated to further improvement in outcomes beyond what is achieved with prompt revascularization in the setting of cardiogenic shock [5,6]. Therefore, utilization of percutaneous MCS devices to augment cardiac output and decrease LV filling pressures by LV unloading may act as a successful adjunct to PCI as a bridge to myocardial recovery in these critically ill patients [24].

## 4. Hemodynamic Effects of Percutaneous Mechanical Circulatory Support Devices

**Figure 1:**
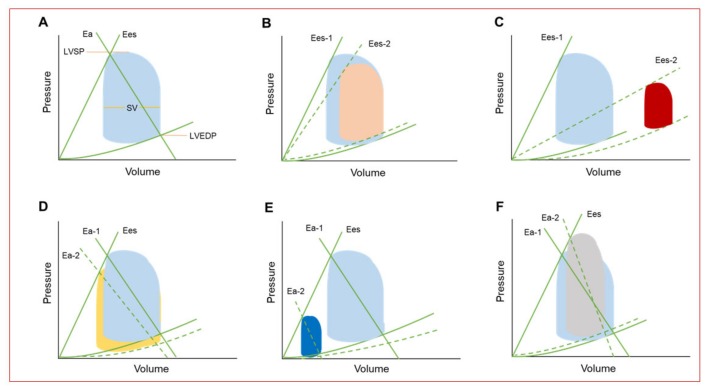
(**A**) Normal pressure-volume (PV) loop. Effective arterial elastance (Ea) is the slope of the line extending from the left ventricular (LV) end-diastolic pressure-volume point through the end-systolic pressure-volume point of the loop. Ea is determined by the total peripheral resistance and heart rate and gives an estimate of the LV afterload. End-systolic elastance (Ees) is the slope of the line extending from the volume-axis intercept V_0_ through the end-systolic pressure-volume point of the loop and represents the ventricular contractility. The width of the PV loop represents stroke volume (SV), which can be extracted by calculating the difference between the end-diastolic and end-systolic volumes. (**B**) PV loop in the setting of acute myocardial infarction (AMI) showing decreased contractility (Ees) and SV in addition to increased LV end-diastolic pressure (LVEDP). (**C**) PV loop of patients with cardiogenic shock showing severe reduction in contractility (Ees) and SV in addition to markedly increased LVEDP and LV end-diastolic volume (LVEDV). (**D**) Illustration of PV loop change after intraaortic balloon (IABP) counterpulsation showing mildly reduced LVEDP and LV end systolic pressure (LVESP) resulting in modest afterload (Ea) reduction and increase in SV. (**E**) PV loop with percutaneous LV assist device support (Impella or TandemHeart) showing marked reduction in LVEDP, LVESP, and SV, with a net effect of substantial afterload, preload, and LV workload reduction. (**F**) LV loop with veno-arterial extracorporeal membrane oxygenation (VA-ECMO) without LV venting increases LVEDP and LVESP, while reduces stroke volume and an ultimate increase in afterload (Ea) and LV loading.

The hemodynamic condition in steady state as well as in various cardiac abnormalities is illustrated by the pressure-volume loop, which provides fundamental information to the understanding of the underlying hemodynamic imbalance and the anticipated effect with the use of each type of the available MCS devices (Figure 1) [11,18]. Pressure-volume loops not only provides a platform for explaining ventricular mechanics, such as contractile and relaxation properties, stroke volume, and cardiac work, but provides a platform for understanding the determinants of myocardial oxygen consumption represented mainly by LV work [11,18,25,26]. Each one of these hemodynamic variables can be compromised based on the clinical presentation. In AMI, for example, patients may mainly present with decreased myocardial contractility and stroke volume in addition to increased myocardial oxygen demand and diminished coronary blood flow (Figure 1B). In cardiogenic shock, LV contractility and stroke volume are severely reduced, while LV end-diastolic volume (LVEDV) and pressure (LVEDP) as well as myocardial oxygen demand are considerably increased (Figure 1C). In the setting of mechanical support, the change in the volume-pressure loops is dependent on the type of the MCS device and the amount of support [18,27,28]. IABP provides modest hemodynamic support demonstrated by modest reduction in both LV systolic and diastolic pressures with afterload reduction and increase in stroke volume (Figure 1D). Percutaneous LV assist devices (including Impella and TandemHeart) result in remarkable reduction in LV systolic and diastolic pressures, LV volumes, and stroke volume resulting in significant decrease in LV work. Unlike the other forms of support, continuous pumping of blood directly from the LV by Impella is not dependent on blood ejection through the aortic valve and LV unloading can, therefore, be augmented by increasing the flow rate, thus resulting in further reduction in LV filling pressures and in myocardial oxygen demand (Figure 1E). Venoarterial extracorporeal membrane oxygenation (VA-ECMO) has the capacity to assume responsibility for the entire cardiac output providing biventricular support in combination with full gas exchange. Strictly on a hemodynamic basis and without an LV venting strategy, use of VA-ECMO results in increased LV systolic and diastolic pressures and reduced stroke volume, with a final flow-dependent increase in afterload and LVEDP (Figure 1F). Therefore, LV venting assisted by Impella or IABP may be ultimately required to mitigate LV loading and decrease left-sided filling pressures and myocardial oxygen demand, especially when the aortic valve remains persistently closed during ECMO support indicating a maximal LV loading condition.

## 5. Available Percutaneous Mechanical Circulatory Support Devices

Several percutaneous MCS devices are currently available to assist interventional cardiologists during high-risk PCIs both for patients undergoing a complex PCI and for those requiring PCI in the setting of cardiogenic sock complicating AMI. Types of available percutaneous MCS devices and comparisons of their characteristics and hemodynamic impact are presented in Table 2 [29,30]. Generally, the goal of MCS devices is to improve cardiac power (defined as the product of mean arterial blood pressure and cardiac output), which has been demonstrated to be a strong predictor of outcomes in patients with cardiogenic shock [31,32]. Therefore, each device has its unique impact on cardiovascular hemodynamics, but an ideal MCS device should ultimately provide circulatory support to achieve adequate systemic tissue perfusion, by increasing mean arterial pressure, while concurrently decreasing myocardial oxygen demand, by reducing both LV volume (preload) and pressure (afterload).

### 5.1. Intraaortic Balloon Bump

Since its introduction in the 1960s, IABP remains the most widely used device for temporary support in hemodynamically unstable patients due to its greater availability and ease of insertion as compared to other temporary devices [33]. It is typically inserted via femoral arterial access, though axillary or subclavian approaches are also feasible and have the advantage of enabling ambulation among stabilized patients. IABP support is driven by electrocardiogram (ECG)-guided balloon inflation with helium (due to its low viscosity that facilitates easy transfer in and out of the balloon in addition to its rapid absorption in blood in case of balloon rupture) at the onset of diastole and deflation at the onset of LV systole. This diastolic pressure augmentation results in increased coronary perfusion, decreased afterload, decreased cardiac work, and decreased myocardial oxygen demand. Despite these favorable effects, the increase in cardiac output is usually minimal and it may not, therefore, provide adequate support to improve end-organ perfusion in patients with severe cardiogenic shock. Moreover, optimal support is dependent on the underlying heart work and also on other factors, such as a stable electrical rhythm, optimal balloon position and sizing, and the timing of balloon inflation in diastole and deflation in systole [34].

The complications associated with IABP use are mainly vascular, including limb ischemia, vascular injury, and stroke [35]. Although it rarely occurs, trauma to the aorta or ostia of the visceral arteries can result in life-threatening complications, including acute renal failure, bowel ischemia, and atheroembolic events. Other complications include bleeding, infection, and thrombocytopenia. Anticoagulation therapy (usually with heparin) is generally recommended for patients supported with an IABP to prevent ischemic complications though no definitive evidence exists to support this approach and some centers do not use anticoagulation with 1:1 pumping, particularly in patients at high bleeding risk. IABP is contraindicated in patients with severe peripheral arterial or aortic disease and in those with moderate or severe aortic valve regurgitation. With the emergence of newer short term support strategies that provide greater hemodynamic support and ventricular unloading, the role of IABP support in the setting of cardiogenic shock or high risk PCI will continue to decline as experience grows with more promising short-term MCS therapies. According to the current European Society of Cardiology (ESC) guidelines, IABP is not indicated for patients with cardiogenic shock complicating AMI (Class III) [10], and a recent study has found that IABP is considered one of the medical reversals in clinical practice as this widespread therapeutic strategy bolstered by retrospective studies and previous guidelines was strongly challenged by subsequent large prospective and randomized studies showing no clinical benefit of its utilization in this clinical setting [36].

### 5.2. Impella Devices

The Impella pumps are continuous nonpulsatile microaxial flow devices that are deployed into the LV across the aortic valve and unload the LV by pumping blood from the LV cavity to the ascending aorta. The Impella 2.5 and CP pumps (Abiomed Inc, Danvers, MA, USA) can be placed percutaneously and provide maximal flow rates of 2.5 and 3.0–4.0 L/min, respectively, while the Impella 5.0 and Impella LD (Abiomed Inc, Danvers, MA, USA) devices are larger LV assist axial-flow pumps that require surgical cutdown and provide up to 5.0 L/min of cardiac output [14,37,38]. Similarly, the right-sided Impella RP is designed for right ventricular (RV) hemodynamic support by propelling blood from the inferior vena cava and right atrium (RA) to the pulmonary arteries and can also be deployed percutaneously. The percutaneous Impella devices for LV support are inserted most commonly through the femoral artery and then advanced in a retrograde fashion to the LV with a flexible pigtail loop that stabilizes the pump in the LV chamber and protects from LV perforation. Impella insertion requires large-bore arterial cannulation (13-F for Impella 2.5 and 14-F for Impella CP) and therefore, it is essential to ensure adequate femoral and iliac arterial diameters to enable device delivery via a femoral approach. Alternatively, though less commonly used, axillary or subclavian arterial accesses can be used to deliver these pumps percutaneously. For high-risk PCI, the device is usually removed at the end of the procedure. However, in patients with persistent cardiogenic shock despite revascularization, Impella should be retained for further hemodynamic support, a condition that may pose some challenges and thus requires careful assessment and management in the cardiac care unit. Optimal device functioning is crucial and largely depends on appropriate positioning in the LV cavity, as device migration can lead to low flow, ventricular arrhythmias, and hemolysis [39]. In such cases, bedside transthoracic echocardiography-guided device repositioning is usually successful without the need for fluoroscopy [11].

Unlike IABP, Impella (as other percutaneous ventricular assist devices) functions independently of the remaining LV function and does not require synchronization with the cardiac cycle. Therefore, Impella devices are more helpful than IABP in patients with severely depressed LV function who present with significant arrhythmias. Impella results in effective LV unloading thus resulting in decreased LV filling pressures (LVEDP) and myocardial oxygen consumption, while improving cardiac output, mean arterial pressure, and coronary perfusion [40]. All the hemodynamic parameters, including cardiac output, are more markedly improved with Impella use compared with an IABP. Additionally, the more powerful Impella CP and 5.0 devices provide greater hemodynamic support than Impella 2.5 and thereby are more beneficial in patients with profound cardiogenic shock requiring greater hemodynamic support [30].

Despite the improvement in hemodynamic parameters, device-related complications are not rare and can be clinically meaningful, thereby contradicting the potential hemodynamic benefits that can be achieved with the use of Impella devices in some cases. As with any mechanical support device, common complications associated with Impella include vascular trauma, limb ischemia, and bleeding requiring blood transfusion. Moreover, hemolysis is frequently encountered during Impella support due to mechanical erythrocyte shearing, but usually improves after repositioning the device. Based on the Impella EUROSHOCK Registry [39], utilization of Impella 2.5 in the setting of acute cardiogenic shock was found to be associated bleeding at the vascular access site requiring blood transfusion in 24% of cases and hemolysis requiring blood transfusion in 7.5% of cases after a mean Impella support duration of approximately 48 h. Impella may also worsen right-to-left shunting and hypoxemia among patients with a preexisting ventricular or atrial septal defect. As device technology continues to improve and the Impella-associated complications continue to decrease, the use of Impella devices has been steadily increasing over the last several years [41]. This is also owing to its relative ease of deployment and more efficient hemodynamic support compared with other MCS devices.

Impella should not be used in patients with a mechanical aortic valve or LV thrombus as well as in those with severe peripheral arterial disease or who cannot tolerate systemic anticoagulation therapy. Although severe aortic stenosis and regurgitation are considered relative contraindication, the use of Impella in this setting has been shown to be feasible and may be considered in selected high-risk patients with severe aortic stenosis and cardiogenic shock or those with severe LV dysfunction and CAD who require high-risk PCI and/or balloon aortic valvuloplasty as well as in selected patients who develop hemodynamic collapse during transcatheter aortic valve replacement (TAVR) [42].

### 5.3. TandemHeart

The TandemHeart (CardiacAssist) system is an extracorporeal, centrifugal, continuous flow pump, which is available on the market for left, right, and biventricular failure. For LV support, TandemHeart device is percutaneously inserted to pump blood extracorporeally from the left atrium through a transseptal cannula back into the femoral/iliac artery through an arterial cannula using a centrifugal pump that provides 3 to 5 L/min of continuous flow at 3000 to 7500 rpm, respectively [11,37]. The transseptal inflow cannula is a 21-F and contains a large end-hole and 14 side holes that enable effective blood aspiration from the left atrium while the arterial outflow cannula ranges in size between 15-F and 19-F according to the flow rate via the iliofemoral arterial system [43]. The pump is also FDA-approved and available in market for an oxygenator to be added to the circuit thereby allowing for blood oxygenation with simultaneous LV unloading. As with Impella support, anticoagulation (typically with unfractionated heparin) is required with a recommended activated clotting time (ACT) goal of about 250–300 s prior to device activation.

By propelling blood from the left atrium directly to the arterial system, TandemHeart results in a significantly reduced LV preload, filling pressures, and myocardial oxygen demand, while cardiac output and mean arterial pressures are improved. However, because LV output through the aortic valve competes with the retrograde flow from the device, LV afterload increases as the device support is augmented, which may ultimately result in aortic valve closure requiring LV venting [44,45].

Similar to other percutaneous MCS devices, TandemHeart use may infrequently cause limb ischemia and vascular injury [37]. Additionally, as transseptal puncture is needed to insert a large caliber venous cannula, expertise with this technique is crucial for TandemHeart application in clinical practice, which may limit its widespread use, particularly among inexperienced interventional cardiologists not regularly performing transseptal punctures in their practice. Although rare, unique complications related to the transseptal puncture required for MCS with TandemHeart may occur and include cardiac tamponade, hemolysis, and thrombus or air embolism. Finally, device migration with dislodgement of the left atrial cannula to the RA during patient transport or leg movement may cause significant right-to-left shunt, resulting in severe hypoxemia and hemodynamic collapse. Severe peripheral arterial disease, left atrial thrombus, and profound coagulopathy are considered contraindications for TandemHeart use. Moreover, limited experience exists regarding the use of this device among patients with moderate to severe aortic regurgitation or those with ventricular septal defect [46].

### 5.4. Venoarterial Extracorporeal Membrane Oxygenation (VA-ECMO)

Based on the Extracorporeal Life Support Organization (ELSO) registry data, the number of ECMO devices and the number of centers utilizing ECMO are markedly increasing [47,48]. VA-ECMO provides both circulatory and oxygenation support, and therefore it is ideally used in patients with biventricular failure who develop cardiogenic shock and impaired oxygenation requiring cardiopulmonary resuscitation at the time PCI is initiated [49]. This is unlike venovenous ECMO (VV-ECMO), which is reserved for patients with respiratory failure but without significant cardiac dysfunction. When percutaneously inserted, VA-ECMO bypasses both the right and left side of the heart by draining deoxygenated blood from a central vein or RA with an 18-F to 21-F venous cannula and pumping oxygenated blood, after passing via an extracorporeal membrane oxygenator, into the iliofemoral arterial circulation (14-F to 19-F arterial cannula). The VA-ECMO system provides cardiac flow between 3 and 7 L/min depending on cannula sizes and can potentially be maintained for several days to weeks among patients with persistent cardiogenic shock as a bridge to recovery, permanent LV assist device implantation, or heart transplantation. Due to the substantial hemodynamic support provided by VA-ECMO, the hemodynamic and metabolic derangement resulted from cardiogenic shock is generally corrected within hours of device activation. Unfractionated heparin is typically used during ECMO support but other anticoagulants, such as bivalirudin, particularly in patients with heparin-induced thrombocytopenia who are unable to receive heparin, have been increasingly used as alternatives. The extent of anticoagulation during ECMO support is largely dependent on the type of membrane oxygenator and ranges from 180–250 s [50].

VA-ECMO support results in a remarkable increase in cardiac output and mean arterial pressure. However, its use is limited by retrograde blood flow leading to LV afterload mismatch, inadequate LV decompression, and high myocardial oxygen demand. The concurrent use of IABP or Impella can add further support by direct unloading of the LV and reducing ventricular wall stress [51,52]. Recently, a novel electrocardiogram (ECG)-synchronized, pulsatile VA-ECMO system, labeled i-cor (Xenios AG), has been introduced. The i-cor system consists of an ECG-triggered diagonal pump, which has a feature of diastolic augmentation and a capacity of providing a support up to 8 L/min. The main difference of the i-cor pump is the ability to generate a physiological pulse and to manage rotational timing to synchronize the pulse with the cardiac cycle of the native heart in order to provide adequate and physiologic circulatory support. By decreasing extracorporeal blood flow during systole and increasing flow during diastole, i-cor assist device flow decreases afterload and LV end-diastolic pressure and improves LV function and coronary flow compared with standard continuous VA-ECMO flow [53,54,55]. The first-in-man study, involving 15 patients with cardiogenic shock (71% due to AMI), showed that the i-cor pump was safe and applicable in clinical practice and implicated a hemodynamic benefit [56]. A multicenter study designed to test the safety and feasibility of this innovative pulsatile cardiac-synchronous MCS system in a larger population (the “SynCor” trial) is still ongoing.

One of the recent advancements in the percutaneous extracorporeal life support (ECLS) technology is the development of the Lifebridge B2T “bridge to therapy” system (Zoll Medical GmbH, Köln, Germany), which is a miniaturized and portable heart-lung support system similar in design to standard ECMO systems with an ability to provide cardiovascular stabilization and sufficient end-organ perfusion immediately after circulatory arrest. The Lifebridge system enables rapid application within 5 min due to its automated set-up and portable design in addition to less cumbersome transportation of the patient than standard ECMO equipment due to its smaller size and suitcase configuration. Other technical features include a battery life of 2 h, an overall weight of 18 kg (39.6 lb.), and a maximal blood flow of 6 L/min. Real-world clinical data of the German Lifebridge Registry involving 444 patients from 60 tertiary cardiovascular centers has been recently published [57] showing that this transportable automated ECLS system was safely applicable for hemodynamic stabilization with acceptable complications. However, mortality rates remained extremely high in these critically ill patients with immediate survival rates of 36% and 16% at 30-days after device implementation, especially in those with high lactate levels on admission.

Adverse events related to VA-ECMO include bleeding (with excessive anticoagulation), thromboembolic events in the circuit or systemically (if anticoagulation is inadequate), cannula-induced vascular injuries, infection, stroke (ischemic or hemorrhagic), hemolysis, and limb ischemia [58]. To reduce the risk of limb ischemia, a second antegrade arterial sheath can be inserted into the superficial femoral artery and when fed by the main arterial cannula can provide secured antegrade perfusion to the limb. Contraindications to peripheral VA-ECMO include patients with severe chronic organ dysfunction (renal failure, cirrhosis, or emphysema), prolonged cardiopulmonary resuscitation without adequate tissue perfusion, severe peripheral arterial disease, and patients unable to receive anticoagulation [11].

### 5.5. Other Percutaneous Mechanical Support Devices

Percutaneous LV MCS pumps under investigation include the pulsatile iVAC 2L (PulseCath BV, Arnhem, The Netherlands), which has been recently evaluated prospectively in a pilot study of 14 patients undergoing high-risk PCI demonstrating 100% angiographic success [59]. The Aortix (Procyrion, Houston, TX, USA) and Reitan (Cardiobridge, Hechingen, Germany) devices are other investigational devices that are deployed in the descending aorta similar to the IABP.

Apart from LV support, RV failure refractory to medical therapy is increasingly becoming a clinical challenge, thereby prompting the development of devices to specifically provide RV support. Large inferior AMI may cause predominant RV failure with cardiogenic shock with or without severe LV dysfunction. When cardiogenic shock is persistent despite maximal medical therapy, the options for MCS in this setting include VA-ECMO, surgical implantation of RVAD or total artificial heart (TAH), and heart transplantation [60]. The recent development of Impella RP launched an evolving field of percutaneous mechanical therapies for refractory RV failure. Impella RP is an intracardiac microaxial pump designed predominantly for management of primary RV failure, particularly in the setting of AMI, and can be inserted through the femoral vein to eject blood from the inferior vena cava directly to the pulmonary artery. The safety and reliability of the RP Impella has been established in the prospective RECOVER RIGHT study for severe isolated RV dysfunction [61]. Like LV Impella devices, complications that may occur with RP Impella support include bleeding, thrombosis, hemolysis, or infection.

## 6. Clinical Benefit of Percutaneous MCS Devices for PCI

The most recent clinical practice guidelines regarding the use of percutaneous MCS for PCI and management of ACS recommend consideration of the use of these devices in the setting of high-risk PCI and AMI complicated by cardiogenic shock [11]. However, despite accumulating evidence of hemodynamic improvement using various MCS devices, a convincing clinical benefit based on randomized controlled trials has not yet been demonstrated among this population. Because this is an extremely high-risk group of patients, improving clinical outcomes can be challenging and further research is still necessary to examine the optimal device features, the timing of support, and the appropriate patient for achieving maximal clinical benefit from these devices during PCI. The salient findings of contemporary studies examining the effect of MCS devices on outcomes in cardiogenic shock complicating AMI and high-risk PCI are summarized in Table 3 and Table 4, respectively.

### 6.1. Intraaortic Balloon Bump

In the setting of PCI with cardiogenic shock complicating AMI, a previous retrospective study has suggested a potential benefit with early IABP support placed prior to PCI showing decreased rates of in-hospital mortality and cardiac adverse events compared with IABP placed only following PCI [62]. In a meta-analysis by Sjauw et al. [63], no mortality benefit or improvement in LV ejection fraction were found with utilizing IABP among patients undergoing PCI for ST-elevation myocardial infarction (STEMI), while higher stroke and bleeding rates were observed in the IABP group. Furthermore, among patients with cardiogenic shock undergoing PCI, there was a 6% increase in 30-day mortality [63]. Not only were randomized studies analyzed but cohort studies were also included in this meta-analysis; therefore, it could be subject to selection bias as sicker patients with more profound cardiogenic shock were more likely to be supported with IABP. In the SHOCK trial [64], IABP was not been shown to reduce mortality or major adverse events in patients with cardiogenic shock or high-risk PCI except in patients with STEMI. Subsequently, the IABP-SHOCK II trial [5], a prospective randomized controlled trial designed to study the effect of IABP involving 600 patients with AMI and cardiogenic shock undergoing early revascularization, showed no improvement in survival with IABP at 30 days [5] and subsequently at 1 year [65] and 6 years [66] post AMI. Moreover, there were no significant differences in any of the secondary clinical and laboratory (including lactate and creatinine) endpoints, and there were no significant differences in subgroup analyses. Based on the current guidelines, IABP is largely recommended in patients with mechanical complications post AMI or during transport of unstable patients from PCI centers without to centers with on-site cardiac surgery [12,67].

Among patients undergoing PCI without evident cardiogenic shock, IABP use was tested in the CRISP-AMI (Counterpulsation to Reduce Infarct Size Pre-PCI Acute Myocardial Infarction) trial [68]; a 30-center randomized controlled trial involving 337 patients with anterior STEMI, which found that routine IABP placement immediately prior to PCI had no significant effect on the infarct size as assessed by magnetic resonance imaging 3 to 5 days post PCI or on survival rates after 6 months of follow-up. Similarly, in a nonrandomized study using the National Cardiovascular Data Registry database, IABP utilization in high-risk PCI was not associated with lower mortality, and wide regional variations in the use of IABP was noted among the different centers [69]. Finally, the BCIS-1 trial [3], a prospective randomized controlled trial involving 301 patients randomized to routine IABP versus provisional IABP support for high-risk PCI, found no significant differences in mortality between the two groups. However, a long-term follow-up of more than four years showed a 34% relative reduction in all-cause mortality risk with routine IABP in patients with severe ischemic cardiomyopathy undergoing high-risk PCI, which might be attributed to lower incidence of procedural hypotension with preplanned IABP insertion [70].

Given these conflicting results and the controversy surrounding its benefit, the use of IABP in the setting of STEMI complicated with cardiogenic shock is decreasing and its routine use has been recently downgraded to class III (harm) by the ESC [10] and to class IIa (should be considered) by the American Heart Association (AHA)/American College of Cardiology (ACC) [71] guidelines. Furthermore, due to the introduction of new devices, such as Impella, and the continuous advancement in MCS technology, which provides superior hemodynamic support and potentially more favorable outcomes, the future role of IABP in management of cardiogenic shock and ventricular unloading may continue to decline.

### 6.2. Impella Devices

There are currently limited available data to establish a significant clinical benefit of Impella use in patients with cardiogenic shock undergoing PCI. Impella devices provide greater hemodynamic support with a more pronounced cardiac output augmentation and LV unloading than IABP. In 2008, the ISAR-SHOCK (Impella LP 2.5 versus IABP in Cardiogenic SHOCK) trial [72] was the first randomized clinical study to assess the safety and efficacy of Impella 2.5 in the setting of AMI complicated by cardiogenic shock as compared to IABP. Twenty-five patients were randomized to the Impella 2.5 LP device or IABP, and the primary endpoint was a change in cardiac index after 30 min of support. All patients received PCI of the infarct-related artery and remained in shock. Patients supported with an Impella had greater increase in cardiac index and mean arterial blood pressure after 30 min of support. However, there was no difference in mortality or in the rates of bleeding or limb ischemia between the two groups [72]. Subsequently, the safety and efficacy of Impella 2.5 LP was examined in the EUROSHOCK multicenter registry [39] involving 120 patients with severe cardiogenic shock refractory to conventional therapy, among which cardiopulmonary resuscitation was performed in more than 40%, showing high overall in-hospital mortality rates reaching 64% without survival benefit in the group treated with an Impella. Similarly, the IMPRESS in Severe SHOCK (IMPella versus IABP Reduces mortality in STEMI patients treated with primary PCI in Severe cardiogenic SHOCK) trial [6] enrolled 48 mechanically ventilated patients with severe cardiogenic shock after AMI who were randomized 1:1 to Impella CP or IABP and followed for all-cause mortality at 30 days and 6 months post AMI. Importantly, 44 (92%) patients had cardiac arrest prior to randomization with interquartile time till return of spontaneous circulation ranging from 15 to 52 min and a considerable proportion of patients (46%) died due to anoxic brain damage. The overall mortality in the study was 50% and there were no significant differences between the two arms (50% versus 46% at 30 days and 50% versus 50% at 6 months in the IABP and Impella CP groups, respectively), thus reflecting a very high risk cohort presenting with late-stage cardiogenic shock [6,73]. The only study reporting a mortality benefit of Impella in the setting of cardiogenic shock complicating AMI was by Karatolios et al. [74], who conducted a retrospective, single-center study including 90 patients suffering from AMI and cardiogenic shock treated with Impella (n = 27) or medical treatment alone (n = 63). Patients in the Impella group were sicker, evidenced by higher lactate levels, longer low cardiac output duration, and lower LV ejection fraction than those treated medically. When 20 patients of each group were matched, patients supported with Impella had decreased rates of in-hospital (35% versus 80%; *p* = 0.01) and 6-month (40% versus 80%; *p* = 0.02) mortality [74]. More recently, using IABP-SHOCK II trial inclusion and exclusion criteria, a retrospective analysis of 237 patients with AMI and cardiogenic shock treated with Impella 2.5 (~30% of patients) or Impella CP (~70% of patients) were propensity matched to the same number of patients from the IABP-SHOCK II trial [75]. There was no significant difference in 30-day all-cause mortality. Moreover, severe or life-threatening bleedings as well as peripheral vascular complications occurred more often in the Impella group. Limiting the analysis to IABP-treated patients as the control group showed comparable results with no evidence of favorable outcomes with Impella use [75]. Finally, a meta-analysis including 588 patients from the main aforementioned studies, the use of MCS with Impella in the setting of AMI and cardiogenic shock was not associated with improved short-time survival but there were higher rates of complications when compared with IABP and medical treatment [76]. It is important to recognize, however, that the vast majority of patients included in these studies were in profound cardiogenic shock and after cardiac arrest. As the use of Impella in patients with less severe shock or pre-shock conditions was not the focus of these studies, its effect on outcomes cannot, therefore, be addressed based on the current data and further studies are still warranted in these settings.

The effectiveness and safety of Impella support for planned high-risk coronary interventions have been investigated in small studies showing encouraging results. In the AMC MAC1 study [77], 19 consecutive high-risk and poor surgical candidates with moderate-to-severe LV dysfunction (ejection fraction < 40%) underwent PCI of an unprotected left main or the last remaining vessel with Impella 2.5 support showing 100% procedural success and no important device-related adverse events, thereby demonstrating safety and feasibility of utilizing Impella devices for high-risk PCI. This has also been confirmed in the PROTECT I trial [78], a prospective and multicenter study, which showed that Impella 2.5 system is safe, easy to implant, and provides excellent hemodynamic support for a mean duration of 1.7 h (range: 0.4–2.5 h) during high-risk PCI. The real-world use of the Impella 2.5 in complex high-risk PCI showed an angiographic revascularization success of 99% in the overall cohort and in 90% of patients with multivessel revascularization. Survival rates were 91% and 81% at 6 months and 12 months, respectively, despite including inoperable patients with a high prevalence of LV dysfunction, New York Heart Association (NYHA) class III and IV heart failure, and chronic renal dysfunction [14].

The largest prospective randomized clinical study to examine the effect of hemodynamic support with Impella in patients undergoing high-risk PCI was the PROTECT II trial [4]. In this multi-center study, 452 patients with complex three-vessel disease or unprotected left main CAD and severely depressed LV function were assigned to IABP or Impella 2.5 during non-emergent high-risk PCI. The composite primary endpoint of 30-day incidence of 11 major adverse events was similar between the Impella and IABP groups (35.1% for Impella 2.5 versus 40.1% for IABP, *p* = 0.227 in the intention-to-treat population, and 34.3% versus 42.2%, *p* = 0.092 in the per-protocol population). Impella did provide greater hemodynamic support and at 90-day follow-up there was a trend towards a decreased incidence of adverse events with Impella in the intention-to-treat population (40.6% versus 49.3%, *p* = 0.066) and significantly decreased events in the per-protocol population (40.0% versus 51.0%, *p* = 0.023) [4]. A subsequent analysis of the outcomes using a prognostic ally important definition of AMI based on new Q-waves or >8× increase in creatinine kinase-MB, demonstrated that that Impella resulted in improved event-free survival after 90 days of follow-up, supporting a late benefit that could be attributed to more stable procedural hemodynamics that facilitate the performance of more complete revascularization and complex PCI procedures, such as rotational atherectomy and bifurcation coronary disease intervention, more safely [79]. A more recent consecutive real-world cohort of high-risk PCI patients, new generation MCS devices (including Impella CP, Heartmate PHP, and PulseCath iVAC2L) have demonstrated a significant reduction in the composite endpoint of serious peri-procedural adverse events, including cardiac arrest and 30-day mortality despite worse LV function and higher Synergy between Percutaneous Coronary Intervention with TAXUS and Cardiac Surgery (SYNTAX)-I score observed in patients protected with MCS [8]. Interestingly, patients under age of 75, with a SYNTAX-I score > 32, and with an LV ejection fraction < 30% derived most potential benefit from MCS utilization during PCI. These promising findings indicate that the more powerful LV support obtained using new generation MCS devices may be necessary for improving clinical outcomes, which has not been evidently seen using the IABP or Impella 2.5 devices.

### 6.3. TandemHeart

Small studies have shown a significant improvement in hemodynamics with TandemHeart use, but were underpowered to show improvement in survival. In an observational study by Kar et al. [43], TandemHeart placement in patients with severe cardiogenic shock refractory to conventional inotropic or IABP therapy was associated with improvement in cardiac index (increased from 0.5 to 3 L/min/m^2^), PCWP (decreased from 31 to 17 mmHg), and mixed venous oxygen saturation (increased from 49 to 69%), as well as improvement in kidney function (urine output increased from 70 to 1200 mL/day and creatinine decreased from 1.5 to 1.2 mg/dL) and lactic acid level (decreased from 11 to 1.5 mg/dL). However, overall mortality (40.2% at 30 days and 45.3% at 6 months) and bleeding complications remained high [43]. In a small randomized study involving patients in cardiogenic shock after AMI, with intended PCI to the infarcted artery, who were assigned to either IABP (n = 20) or TandemHeart (n = 21), hemodynamic and metabolic parameters was more effectively reversed by TandemHeart than by IABP treatment. However, adverse events, such as severe bleeding and limb ischemia, were encountered more frequently after TandemHeart support, and 30-day mortality was similar [80]. Similarly, a small randomized trial of patients presenting within 24 h of the development of cardiogenic shock and assigned to IABP or TandemHeart showed superior hemodynamic parameters with TandemHeart, even in patients failing IABP, but no significant differences in in-hospital mortality or severe adverse events when compared with IABP alone [81].

Data on the use of TandemHeart in high-risk PCI is limited to observational studies. A study from the Mayo Clinic summarized data on 54 consecutive patients undergoing high-risk PCI using a TandemHeart device for support, demonstrating feasibility and safety of this device to allow performance of high-risk and complex intervention [82]. All patients were deemed high risk for surgery and underwent complex PCI, with a Society of Thoracic Surgery (STS) mortality risk score of 13%, a median SYNTAX score of 33, and the majority of patients underwent left main and multivessel PCI. Procedural success was achieved in 97% of cases, with hemodynamic improvement during the procedure, and 6-month survival was 87%. However, major vascular complications occurred in 13% of cases [82]. Additional small series of patients undergoing TandemHeart-assisted high-risk PCI have shown comparable results. A meta-analysis of usefulness of percutaneous MCS devices, including eight cohort studies with 205 patients supported by TandemHeart and 12 studies with 1346 patients supported with Impella 2.5 during high-risk PCI, found 30-day mortality rates of 8% and major bleeding rates of 3.6% with TandemHeart compared with 3.5% and 7.1% with Impella 2.5, respectively. Overall periprocedural outcomes were comparable between the two groups [83].

### 6.4. Venoarterial Extracorporeal Membrane Oxygenation (VA-ECMO)

While ECMO provides excellent cardiopulmonary support with a relative ease of implementation, its use in the setting of AMI and shock is limited by the need for specialized perfusion expertise and nursing as well as the possibility for increased LV stroke work and myocardial oxygen demand, which can precipitate further myocardial ischemia. Additionally, there is need for large bore cannulas and aggressive antithrombotic therapy which increase the risk of bleeding. A recent single-center study [84] reported a 67% survival to discharge rate among 18 consecutive patients who received femoral VA-ECMO in the cardiac catheterization lab for severe shock due to ACS. The average length of ECMO support was 3.2 days and 17 (94%) patients required at least one blood transfusion with higher bleeding rates observed among those treated with glycoprotein IIb/IIIa inhibitors [84]. In another small retrospective study [85], a total of 15 patients undergoing VA-ECMO placement for AMI with refractory cardiogenic shock were analyzed. One-third of patients presented with out-of-hospital resuscitation and 60% had IABP placed for LV venting. The survival rate after 30 days was 47% and vascular complications occurred in 53% of patients. There is a growing utilization of VA-ECMO in patients suffering from cardiac arrest requiring prolonged cardiopulmonary resuscitation (CPR) showing acceptable survival rate and outcome [86]. A meta-analysis including 1866 patients treated with VA-ECMO for cardiogenic shock or cardiac arrest showed an approximate overall survival of 30% and significant associated morbidity with the performance of this intervention [58]. These data suggest that use of VA-ECMO should be individualized based on risk-benefit analysis derived from vascular anatomy and comorbidities to maximize clinical benefit. Finally, LV unloading during VA-ECMO treatment for cardiogenic shock appears beneficial either with predominant use of IABP [51] or Impella [87] demonstrating significant improvement in survival with the two unloading tools.

## 7. Recommendations for MCS Use During PCI

**Figure 2:**
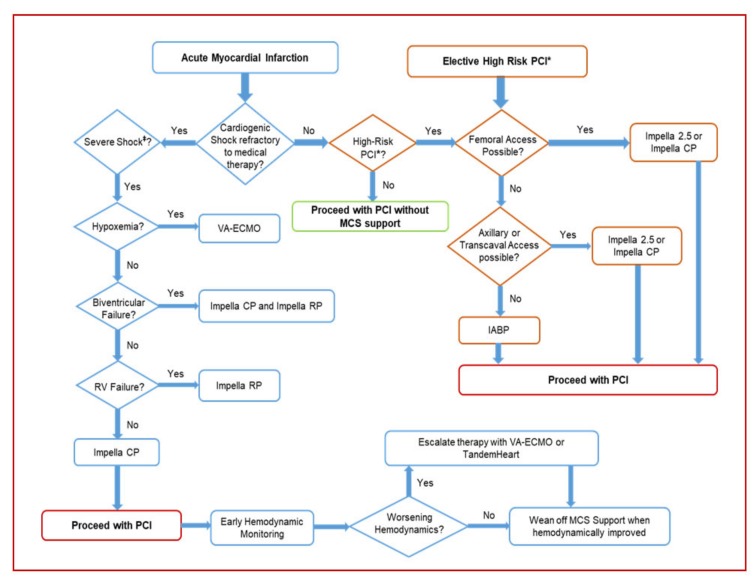
Suggested algorithmic approach for MCS in patients with cardiogenic shock complicating AMI and high-risk PCI. MCS, mechanical circulatory support; AMI, acute myocardial infarction; PCI, percutaneous coronary intervention. IABP, intraaortic balloon bump; VA-ECMO, venoarterial extracorporeal membrane oxygenation. * High-risk PCI is defined as presented in Table 1 and mainly include comorbidities, severe LV dysfunction (EF < 35%), and complex coronary artery disease involving a large territory, such as unprotected left main, sole-remaining vessel, or three-vessel disease. ^‡^ Severe cardiogenic shock is defined as markedly abnormal hemodynamic parameters (systolic blood pressure < 90 mmHg, heart rate > 120 beats per minute, cardiac index < 1.5 L/min/m^2^, pulmonary capillary wedge pressure/left ventricular end-diastolic pressure > 30 mmHg), metabolic (lactate > 4 mg/dL), and clinical (confusion, cool extremities, on ≥2 vasopressors/inotropes) parameters.

A suggested algorithmic approach to MCS use during PCI for cardiogenic shock complicating AMI and high-risk PCI without cardiogenic shock is depicted in Figure 2. Interventional cardiologists are challenged with a growing number of patients in need of coronary revascularization but who are hemodynamically unstable or deemed poor surgical candidates and too high risk for PCI. The use of percutaneous MCS devices has been proven to be feasible and safe in clinical conditions previously considered for conservative therapy only. However, data on survival benefit as a result of utilizing these percutaneous devices is still lacking. It is not therefore surprising that there has been a modest adoption of these devices in the current practice, reflecting the equivocal results from the current evidence base in addition to the uncertainty as to which patients MCS will add a real benefit. Optimal timing of device insertion is also an important variable. In light of the currently available data, it is reasonable that utilization of MCS devices in clinical practice should be advocated in an individualized manner based on a detailed review of the risks and benefits rather than as a standard of care for every complex procedure. The recommendations on the use of a specific MCS device are based on the anticipated hemodynamic effects and risks as well as clinical outcome data. Based on the recent SCAI/ACC/HFSA/STS Clinical Expert Consensus Statement published in 2015 [11], percutaneous MCS may be considered in carefully selected patients with severe cardiogenic shock complicating AMI unresponsive to pharmacologic support as a bridge to recovery following PCI or definitive therapy. The evidence is stronger in the setting of mechanical complications post MI, such as ischemic mitral regurgitation when hemodynamic derangement is usually acute and more substantial and the benefit from these devices is more pronounced. There is an increasing indication for temporary MCS use during and after primary PCI for patients presenting with large AMI causing severe LV dysfunction, and RV mechanical support with Impella RP can be considered for RV infarction complicated by cardiogenic shock. Early insertion of MCS devices is essential to attenuate the sequelae that may result from persistent cardiac ischemia and systemic hypoperfusion. The type of MCS device is dependent on multiple factors including the amount of hemodynamic support needed and the ultimate goal of support as well as technical characteristics that should be taken into consideration, such as the ease or deployment, and availability of these devices. IABP is more often used due to the ease of insertion and availability although the benefit of its utility is questionable in patients with AMI and cardiogenic shock. Impella 2.5 or CP provide more powerful hemodynamic support and can be inserted as rapidly as an IABP in experienced centers and are thus considered more favorable devices in appropriate patients. Finally, TandemHeart, VA-ECMO, or Impella 5 (which requires surgical cutdown for delivery) should be reserved for patients who continue to deteriorate despite such support. In patients who present with biventricular or cardiopulmonary failure, VA-ECMO is recommended as the first choice. For isolated RV failure with cardiogenic shock, Impella RP is an available percutaneous MCS option that may be considered in such cases [28].

For prophylactic percutaneous MCS use in the setting of high-risk PCI, great emphasis should be on identifying the patient’s anatomic, hemodynamic, and procedural features that indicate adjunctive MCS support may be necessary and also determine the optimal device to utilize [2]. The current recommendation is to consider using MCS devices in patients with severe LV dysfunction (EF < 35%) or recent presentation with decompensated heart failure in the setting of complex coronary artery disease involving a large territory, such as unprotected left main, sole-remaining vessel, or three-vessel disease. Generally, patients with anticipated noncomplex PCI may be considered for IABP as the first-line MCS option with Impella as back up support, whereas those with anticipated technically challenging or prolonged procedure (rotational atherectomy or bifunctional stenting) may be considered for Impella, TandemHeart, or VA-ECMO depending on vascular anatomy, RV function, expertise, and availability [2,88]. The more severe the clinical and anatomic circumstances, the greater the potential benefit of MCS use [11]. MCS support should be initiated before the start of the PCI and in most cases can be removed immediately after the intervention. In cases with hemodynamic derangement after PCI, prolonged MCS support should be considered until hemodynamic improvement. Continuous hemodynamic monitoring with pulmonary arterial catheter in encouraged as early as possible to tailor therapy and help with determining the amount and duration of MCS support needed. Finally, the use of MCS devices in the emergent setting has been suggested to be cost-effective compared with surgical ECMO or ventricular assist device support [89], as well as in the elective setting when compared with IABP [90].

## 8. Future Directions

The majority of studies involving the new MCS devices for cardiogenic shock and high risk PCI have demonstrated superior hemodynamic improvement as compared to IABP but these studies were largely underpowered to find mortality benefit and therefore should be regarded as feasibility trials and a basis for larger clinical trials in the future. The DanGer Shock study [91] is an ongoing prospective randomized multicenter study with planned enrollment of 360 patients with AMI complicated by cardiogenic shock randomized 1:1 immediately after shock diagnosis to either Impella CP prior to PCI or current guideline-driven therapy. Patients with coma after out of hospital cardiac arrest are excluded. The primary endpoint is all-cause mortality at 180 days. The DanGer trial will be the first adequately powered randomized controlled trial to examine whether MCS with Impella CP improves survival among patients with cardiogenic shock complicating AMI and will therefore provide fundamental knowledge on the use of transvalvular LV unloading in this setting [91].

Despite the lack of current evidence, we believe that advanced MCS devices may improve clinical outcomes, including survival, in a selected group of patients and in specific circumstances. Future studies should largely focus on three major considerations when examining the clinical benefit of MCS. First, the optimal MCS device design and amount of support needed to achieve hemodynamic as well as metabolic improvement is unclear. For instance, in the IMPRESS in Severe Shock trial, one third of the patients died due to refractory cardiogenic shock and lactate levels remained high in many patients while being on Impella CP support, suggesting that the actual level of support was not sufficient [6,73]. Second, appropriate patient selection is a key factor that should be emphasized in future studies. Because utilization is currently determined based on subjective criteria, many patients may survive without MCS while others with irreversible anoxic brain injury from prolonged shock may not survive even with adequate hemodynamic support. Additionally, patients with shock and multiorgan dysfunction are at significantly higher risk of bleeding due to increased inflammation and coagulopathy which may progress to disseminated intravascular coagulation. Therefore, studies focusing on identifying patients are higher risk of device-associated complications among whom the harm of these complications may outweigh the benefit is clinically meaningful for mitigating device complications. For example, lactate has been shown to be an important biomarker for risk stratification in the setting of cardiogenic shock. Among patients undergoing extracorporeal cardiopulmonary resuscitation due to cardiac arrest, lactate clearance was the sole predictor of neurological outcome as assessed by the Glasgow Outcome Scale (GOS) [92] and in patients with refractory cardiogenic shock requiring VA-ECMO support, lactate levels and lactate clearance during ECMO therapy were predictive markers for 30-day mortality [93]. In patients with cardiac arrest, lactate levels prior to ECMO initiation was significantly associated with increased risk of all-cause mortality at 90 days. Interestingly, lactate was found to be more predictive of outcome than duration of cardiopulmonary resuscitation or absence of return of spontaneous circulation [94]. These findings suggest that early metabolic assessment by measuring plasma lactate levels may be an important prognostic marker for risk stratification when considering MCS in patients with cardiogenic shock. One study has shown that high body mass index (BMI) was an additional predictor of 30-day mortality in patients with AMI and cardiogenic shock supported by VA-ECMO [95]. However, the influence of BMI on outcomes among patients supported by other MCS devices is unclear.

Third, the timing of MCS device insertion in relation to primary PCI among patients with AMI and cardiogenic shock can be critical to achieve survival benefit and future studies should focus on determining the optimal timing of MCS, which remains elusive. Recent studies have shown in an animal model that mechanical support with LV unloading reduces infarct size and favorably mediates key biological pathways associated with inflammation and maladaptive cardiac remodeling when mechanical support is initiated prior to coronary reperfusion compared with early reperfusion without support [96,97,98,99]. In a prospective safety and feasibility first-in-human study known as the Door to Unloading With Impella CP System in Acute Myocardial Infarction to Reduce Infarct Size (DTU), LV unloading with the Impella CP with a 30-min delay prior to reperfusion in patient with STEMI is feasible with similar rates of adverse cardiovascular events and 30-day mean infarct size and without prohibitive safety signals [100]. These data suggest that LV unloading prior to PCI initiation in the setting of myocardial ischemia may be pivotal to achieve significant benefit from MCS devices but clinical evidence from randomized control trials is still awaiting. In support of this hypothesis, in the setting of AMI and cardiogenic shock, data from the catheter-based ventricular assist device registry have shown that early MCS implantation (either the Impella 2.5 or Impella CP) before PCI, prior to escalating doses of inotropes or vasopressors and within 75 min from shock onset, was independently associated with improved survival compared with later MCS support [24]. Similarly, data from a quality improvement registry including over 15,000 patients with AMI complicated by cardiogenic shock and supported with Impella have shown wide variation in outcomes across centers with higher survival rates seen when Impella was used as first support strategy, when invasive hemodynamic monitoring was used, and at higher institutional Impella implantation volume [101]. A meta-analysis of 3 available studies on the use of early Impella in patients with AMI and cardiogenic shock found that early initiation of Impella decreased in-hospital or 30-day mortality by 48% compared with late initiation of Impella [102].

Based on these promising retrospective data, and after the FDA’s approval of Impella for cardiogenic shock complicating AMI, investigators have organized the construct of a multicenter national shock protocol now referred to as the “National Cardiogenic Shock Initiative” (NCSI) emphasizing invasive hemodynamic monitoring and rapid initiation of percutaneous MCS support. The analysis of the first 171 patients enrolled in NCSI was recently published [7] and included patients presenting with AMI and cardiogenic shock using the same inclusion and exclusion criteria from the “SHOCK” trial (with an additional exclusion criteria of IABP use prior to MCS). The majority of patients were supported with Impella CP (92%) prior to PCI (74%) with an average door to support of 85 min in STEMI cases. Moreover, right heart catheterization with hemodynamic monitoring was performed in 92% of cases. The use of a protocol-based approach showed improved survival compared with previously reported studies [7]. Despite the limitations of being an observational single arm study, these findings highlight the importance of early MCS prior to PCI for achieving the most benefit from MCS. Future studies are still warranted to confirm these promising findings. Finally, it is necessary to derive efficient risk scores for identifying patients at high risk of developing cardiogenic shock following AMI as this may aid in risk stratification and potential prophylactic utilization of MCS during PCI to mitigate subsequent hemodynamic derangement in this high-risk population [103].

Another field of future research is the percutaneous LV unloading strategy using Impella in combination with VA-ECMO (ECMELLA) to improve outcomes in patients with refractory cardiogenic shock [87,104]. In an all-comers retrospective study, percutaneous LV unloading with Impella on top of VA-ECMO showed improved outcomes, including higher 30-day survival, as compared to predicted outcomes by established risk scores [87]. In addition to the ECMELLA approach, previous studies have also suggested that a combination of VA-ECMO plus IABP is safe and associated with improved in-hospital survival in cardiogenic shock patients [105]. However, given the retrospective nature of these studies, prospective and randomized studies are still warranted to further investigate this therapeutic strategy.

## 9. Conclusions

The use of MCS devices is expanding and several MCS devices are currently available and can provide varying magnitude of hemodynamic support during high-risk and complex PCIs. Small randomized controlled trials have provided conflicting results regarding survival benefit using these devices despite substantial hemodynamic and metabolic benefit. However, given the high morbidity and mortality burden among patients undergoing high-risk and complex PCI, detailed hemodynamic assessment and careful patient selection is mandatory to achieve incremental benefit over early revascularization and pharmacologic therapy with utilizing these devices. Furthermore, the optimal timing and magnitude of hemodynamic support during PCI as well as prevention of device-related complications are all important considerations in future research in this field. Until adequate data supporting MCS use in a broader patient population is available, MCS devices during PCI should be individualized based on multiple factors with a recommended use in patients with the greatest potential benefit and a relatively low risk of device-related complications.

## Figures and Tables

**Table 1:** High-Risk Percutaneous Coronary Intervention Characteristics.

**Patient Characteristics**
Increased age
Comorbidities (diabetes mellitus, chronic lung disease, prior myocardial infarction, peripheral arterial disease, frailty)
Severe LV systolic dysfunction (EF < 20–30%)
Severe renal function impairment (eGFR < 30 mL/min/1.73 m^2^).
**Lesion Characteristics**
Severe three-vessel coronary artery disease
Unprotected left main stenosis
Bifurcation disease or ostial stenosis
High SYNTAX score or type C lesions
Chronic total occlusions
Saphenous vein graft disease
Heavily calcified lesions requiring coronary atherectomy
**Clinical Presentation**
Acute coronary syndrome
Heart failure symptoms (dyspnea, orthopnea, PND, exercise intolerance, peripheral edema)
Arrhythmias (atrial fibrillation with RVR, ventricular tachycardia)
Elevated LV end-diastolic pressure
Severe mitral regurgitation (or other valvular disease)

Abbreviations: EF, ejection fraction; eGFR, estimated glomerular filtration rate; LV, left ventricular; PND, paroxysmal nocturnal dyspnea; RVR, rapid ventricular response; SYNTAX, Synergy between Percutaneous Coronary Intervention with Taxus and Cardiac Surgery.

**Table 2:** Comparison of Technical and Clinical Features of Contemporary Percutaneous Mechanical Circulatory Support Devices.

Features	IABP	Impella 2.5	Impella CP	iVAC 2L	TandemHeart	VA-ECMO
Inflow/outflow	Aorta	LV→aorta	LV→aorta	LV→aorta	LA→aorta	RA→aorta
Mechanism of action	Pneumatic	Axial flow	Axial flow	Pulsatile flow	Centrifugal flow	Centrifugal flow
Insertion approach	Pc (FA)	Pc (FA)	Pc (FA)	Pc (FA)	Pc (FA/FV)	Pc (FA/FV)
Sheath size	7–8 F	13 F	14 F	17 F	Venous: 21 F Arterial: 12–19 F	Venous: 17–21 F Arterial: 16–19 F
Flow (L/min)	0.3–0.5	Max 2.5	3.7–4.0	Max 2.8	Max 4.0	Max 7.0
Pump speed (RPM)	N/A	Max 51,000	Max 51,000	40 mL/beat	Max 7500	Max 5000
Duration of support	2–5 days	6 h–10 days	6 h–10 days	6 h–10 days	UP to 14 days	7–10 days
LV function dependency	+	−	−	−	−	−
Synchrony with the cardiac cycle	+	−	−	−	−	−
LV unloading	+	++	+++	+	+++	−
Afterload	↓	↓	↓	↓	↑	↑↑
MAP	↑	↑↑	↑↑	↑↑	↑↑	↑↑
Cardiac index	↑	↑↑	↑↑↑	↑↑	↑↑↑	↑↑↑
PCWP	↓	↓	↓↓	↓	↓↓	↔
LVEDP	↓	↓↓	↓↓	↓↓	↓↓↓	↔
Coronary perfusion	↑	↑	↑	↑	↔	↔
Myocardial oxygen demand	↓	↓↓	↓↓	↓↓	↓↔	↔
Anticoagulation	+	+	+	+	+	+
Implant complexity	+	++	++	++	+++	++
Management complexity	+	++	++	++	+++	+++
Complications	Limb ischemia, bleeding	Hemolysis, limb ischemia, bleeding	Hemolysis, limb ischemia, bleeding	Hemolysis, limb ischemia, bleeding	Limb ischemia, bleeding, hemolysis	Bleeding, limb ischemia, hemolysis
Contraindications	Moderate-to-severe AR, severe PAD	Severe AS/AR, mechanical AoV, LV thrombus, CI to AC	Severe AS/AR, mechanical AoV, LV thrombus, CI to AC	Severe AS/AR, mechanical AoV, LV thrombus, CI to AC	Moderate-to-severe AR, severe PAD, CI to AC, LA thrombus	Moderate-to-severe AR, severe PAD, CI to AC
CE-certification	+	+	+	+	+	+
FDA approval	+	+	+	−	+	+

Abbreviations: AC, anticoagulation; AoV, aortic valve; AR, aortic regurgitation; AS, aortic stenosis; CI, contraindication; FA, femoral artery; FDA, US Food and Drug Administration; FV, femoral vein; LV, left ventricle; LVEDP, left ventricular end-diastolic pressure; IABP, intraaortic balloon pump; MAP, mean arterial pressure; Max, maximum; PAD, peripheral arterial disease; Pc, percutaneous; PCWP, pulmonary capillary wedge pressure; PS, peripheral surgical; RA, right atrium; RPM, rotations per minute; VA-ECMO, venoarterial extracorporeal membrane oxygenation.

**Table 3:** Main Clinical Studies of Percutaneous MCS devices in AMI with Cardiogenic Shock.

First Author/Study (Ref. #)	N	Study Type	Study Arms	Definition	Primary Endpoint	Salient Findings
**IABP**						
IABP-SHOCK-II [5,65,66]	600	RCT	IABP versus no IABP	AMI with cardiogenic shock (SBP < 90 mmHg for >30 min or need for vasoactive agents, pulmonary congestion, impaired organ perfusion)	30-day, 1-year, 6-year all-cause mortality	No difference in survival at 30 days [5], 1 year [65], and 6 years [66]. No differences recurrent MI, stroke, ischemic comp, severe bleeding, or sepsis.
TACTICs [106]	57	RCT	Fibrinolytic therapy with IABP versus without IABP	AMI with sustained hypotension and heart failure with signs of hypoperfusion	6-month all-cause Mortality	No survival benefit except for patients with Killip III/IV supported with IABP.
Waksman et al. [107]	45	Prospective, nonrandomized	Fibrinolytic therapy with IABP versus without IABP	AMI complicated by cardiogenic shock	In-hospital and 1-year all-cause mortality	In-hospital and 1-year survival improved with IABP after early revascularization with fibrinolytic therapy.
NRMI [108]	23,180	Observational	Fibrinolytic or PCI with IABP versus no IABP	AMI with cardiogenic shock at initial presentation or during hospitalization	In-hospital all-cause mortality	IABP was associated with decreased in-hospital mortality in patients received fibrinolysis but not PCI.
Hariss et al. [62]	48	Observational	IABP prior to PCI versus late IABP	AMI complicated by cardiogenic shock	In-hospital all-cause mortality	Early IABP was associated with decreased in-hospital mortality compared with late IABP.
Sjauw et al. [63]	1009 (RCTs) 10,529 (cohort studies)	Meta-analysis (7 RCTs, 9 cohort studies)	IABP versus no IABP	AMI complicated by cardiogenic shock	30-day all-cause mortality	No survival benefit or improvement in LV ejection fraction with IABP.
**Impella**						
ISAR-SHOCK [72]	25	RCT	Impella 2.5 versus IABP	AMI complicated by cardiogenic shock	Change in the CI at 30 min post implantation	Superior hemodynamics with Impella. Mortality was similar between the two groups.
EUROSHOCK [39]	120	Observational	Impella 2.5	AMI complicated by cardiogenic shock	30-day all-cause mortality	30-day mortality was high at 64% despite improvement in hemodynamic and metabolic parameters with Impella.
IMPRESS in Severe Shock [6]	48	RCT	Impella CP versus IABP	AMI with severe shock (SBP < 90 mmHg or the need for vasoactive agents, and all required mechanical ventilation)	30-day all-cause mortality	Mortality occurred in 50% of patients with no significant survival benefit with Impella.
Karatolios et al. [74]	90	Observational	Impella versus medical therapy	AMI with post-cardiac arrest cardiogenic shock	In-hospital all-cause mortality	Impella group had better survival at discharge and after 6 months despite being a sicker group.
Schrage et al. [75]	237	Observational	Impella 2.5 (~30%), Impella CP (~70%) versus IABP (matched from IABP-SHOCK trial)	AMI with cardiogenic shock (SBP < 90 mmHg for >30 min or need for vasoactive agents, pulmonary congestion, impaired organ perfusion)	30-day all-cause mortality	Impella was not associated with lower 30-day mortality. Severe bleedings and peripheral vascular complications were more common with Impella use.
Wernly et al. [76]	588	Meta-analysis (4 studies)	Impella versus IABP or medical therapy alone	AMI with cardiogenic shock	30-day all-cause mortality	No improvement in short-term survival with Impella. Higher risk of major bleeding and peripheral ischemic events with Impella.
Cheng et al. [109]	100	Meta-analysis (3 RCTs; 1 for Impella versus IABP and 2 for TandemHeart versus IABP))	Impella or TandemHeart versus IABP	AMI with cardiogenic shock	30-day all-cause mortality	No significant differences in 30-day mortality. Improved hemodynamics with Impella and TandemHeart. Higher rates of bleeding with TandemHeart and of hemolysis with Impella.
Alushi et al. [110]	116	Observational	Impella 2.5 (~30%), Impella CP (~70%) versus IABP	AMI with cardiogenic shock	30-day all-cause mortality	No significant differences in 30-day mortality. Impella significantly reduced the inotropic score, lactate levels, and improved LVEF compared with IABP. Higher rates of bleeding with Impella.
**TandemHeart**						
Kar et al. [43]	117	Observational	TandemHeart	Severe cardiogenic shock despite vasopressor and IABP support	30-day all-cause mortality	30-day mortality: 40%. Improvement in hemodynamics refractory to vasopressors and IABP.
Thiele et al. [80]	41	RCT	TandemHeart versus IABP	AMI with cardiogenic shock (CI < 2.1 L/min/m^2^, lactate > 2)	Change in cardiac index	Hemodynamic and metabolic parameters were reversed more effectively by TandemHeart. 30-day mortality was similar. Bleeding and ischemic events were more common with TandemHeart.
Burkhoff et al. [81]	42	RCT	TandemHeart versus IABP	Severe cardiogenic shock (most had AMI and failed IABP)	30-day all-cause mortality	Similar mortality rates and adverse events at 30 days. Superior hemodynamics with TandemHeart.
**VA-ECMO**						
Esper et al. [84]	18	Observational	VA-ECMO	Severe cardiogenic shock due to ACS	Survival to hospital discharge	Survival rates at discharge: 67%. High bleeding rates (94% required blood transfusion).
Negi et al. [85]	15	Observational	VA-ECMO	AMI with severe cardiogenic shock (60% had STEMI and IABP support)	Survival to hospital discharge	Survival rates at discharge: 47%. Vascular complications: 53%.
Nichol et al. [111]	1494 (84 studies)	Systematic review	VA-ECMO	Cardiogenic shock or cardiac arrest	Survival to hospital discharge	Survival to hospital discharge: 50%.
Sheu et al. [112]	Group 1: 115 Group 2: 219	Observational	Group 1: profound shock without ECMO versus group 2: profound shock with ECMO	AMI and profound cardiogenic shock (SBP < 75 mmHg despite IABP and vasopressor support)	30-day survival	ECMO group had higher survival rates: 60.9% versus 28% in non-ECMO group.
Takayama et al. [113]	90	Observational	VA-ECMO	Refractory cardiac shock (AMI in 49%)	Survival to hospital discharge	Survival to hospital discharge: 49%. Bleeding and stroke rates: 26%; and LV distension and pulmonary edema: 18%.

Abbreviations: ACS, acute coronary syndrome; AMI, acute myocardial infarction; CI, cardiac index; IABP, intraaortic balloon pump; IMPRESS in Severe SHOCK, IMPella versus IABP Reduces mortality in STEMI patients treated with primary PCI in Severe cardiogenic SHOCK; ISAR-SHOCK, Impella LP 2.5 versus IABP in Cardiogenic SHOCK; LVEF, left ventricular ejection fraction; MCS, mechanical circulatory support; NRMI, National Registry of Myocardial Infarction; PCI, percutaneous coronary intervention; RCT, randomized controlled study; SBP, systolic blood pressure; STEMI, ST-elevation myocardial infarction; VA-ECMO, venoarterial extracorporeal membrane oxygenation.

**Table 4:** Main Clinical Studies of Percutaneous MCS devices in High-Risk PCI.

First Author/Study (Ref. #)	N	Study Type	Study Arms	Definition	Primary Endpoint	Salient Findings
**IABP**						
BCIS-1 [3]	301	RCT	Elective IABP versus no IABP before PCI	High-risk PCI without cardiogenic shock, LVEF < 30%, severe CAD (jeopardy score > 8)	MACE: Composite of death, AMI, stroke, revascularization at hospital discharge	No reduction in MACE. No difference in survival rates at 6 months. Decreased major procedural complications with planned IABP (mainly hypotension).
Extended BCIS-1 [70]	301	RCT	Elective IABP versus no IABP before PCI	High-risk PCI without cardiogenic shock, LVEF < 30%, severe CAD (jeopardy score > 8)	Long-term All-cause mortality	Elective IABP use was associated with a 34% relative reduction in all-cause mortality at 4 years post PCI.
CRISP-AMI [68]	337	RCT	Elective IABP prior to PCI until at least 12 h post versus no IABP	Acute anterior MI without cardiogenic shock	Infarct size measured by cardiac MRI at 3–5 days post PCI	No reduction in infarct size with IABP use. Survival at 6 months and procedural complications were similar between groups.
NCDR [69]	181,599	Observational	Elective IABP versus no IABP before PCI	LVEF < 30%, severe CAD, including patients with cardiogenic shock	In-hospital mortality	IABP use varied significantly across hospitals. No association with differences in in-hospital mortality.
**Impella**						
Henriques et al. [77]	19	Observational	Impella 2.5	High-risk PCI (elderly, most with prior MI, poor surgical candidates, LVEF < 40%)	Safety and feasibility of Impella use	A 100% procedural success and no important device-related adverse events.
PROTECT I [78]	20	Prospective, nonrandomized	Impella 2.5	High-risk PCI (LVEF < 35%, UPLM disease or last patent vessel)	Safety and feasibility of Impella use	Impella is safe, easy to implant, and provides excellent hemodynamic support during high-risk PCI.
USPella [14]	175	Observational	Impella 2.5	High-risk PCI (severe three-vessel disease or UPLM, mean SYNTAX score 36, low LVEF)	MACE at 30 days	MACE: 8%. 30-day, 6-month, and 12-month survival: 96%, 91%, and 88%, respectively.
PROTECT II [4]	452	RCT	Impella 2.5 versus IABP	High-risk PCI (LVEF < 35%, UPLM, three-vessel or last patent vessel disease)	MACE (a composite of 11 adverse events) at 30 days	30-day MACE was similar between groups (ITT) and trend for lower MACE with Impella (PP). 90-day MACE was similar (ITT) and significantly lower with Impella (PP).
Ameelot et al. [8]	198	Observational	Impella CP, heartmate PHP, or PulseCath iVAC2L versus unprotected PCI	Prophylactic high-risk PCI	A composite of procedure-related adverse events	Lower rates of periprocedural adverse events with Impella devices. 30-day survival was significantly higher with Impella versus unsupported PCI.
**TandemHeart**						
Alli et al. [82]	54	Observational	TandemHeart	Prophylactic high-risk PCI (STS score 13%, SYNTAX score 33, three-vessel and UPLM disease)	6-month survival	6-month survival: 87%. Major vascular complications: 13%.
Briasoulis et al. [83]	205	Meta-analysis (8 cohort studies)	TandemHeart	Prophylactic high-risk PCI	30-day all-cause mortality	30-day mortality: 8%. Major bleeding rates: 3.6%.
**VA-ECMO**						
Teirstein et al. [114]	389 (prophylactic support) 180 (standby support)	Observational	VA-ECMO	High-risk PCI (LVEF < 25%, culprit lesion supplying > 50% of the myocardium)	PCI success rates and major complications rates	Comparable results in the prophylactic compared with the standby VA-ECMO support groups. Patients with extremely low LVEF may benefit more from prophylactic support.
Schreiber et al. [115]	149	Observational	VA-ECMO versus IABP	High-risk PCI (low LVEF and multivessel PCI)	MACE: Composite of MI, stroke, death, CABG	No difference in MACE between VA-ECMO and IABP groups. Higher multivessel PCI success rates with VA-ECMO.

Abbreviations: AMI, acute myocardial infarction; BCIS-1, the Balloon pump-assisted Coronary Intervention Study; CRISP-AMI, the Counterpulsation to Reduce Infarct Size Pre-PCI Acute Myocardial Infarction; CABG, coronary artery bypass grafting; IABP, intraaortic balloon pump; ITT, intention to treat analysis; LVEF, left ventricular ejection fraction; MACE, major adverse cardiac events; MCS, mechanical circulatory support; MI, myocardial infarction; NCDR, National Cardiovascular Data Registry; PCI, percutaneous coronary intervention; PP, per protocol analysis; RCT, randomized controlled study; SYNTAX, Synergy between Percutaneous Coronary Intervention with TAXUS and Cardiac Surgery; UPLM, unprotected left main; VA-ECMO, venoarterial extracorporeal membrane oxygenation.
